# Photonic Generation of Triangular-shaped Waveform Based on External Modulation

**DOI:** 10.1038/s41598-018-21613-5

**Published:** 2018-02-20

**Authors:** Jin Yuan, Tigang Ning, Jing Li, Li Pei, Jingjing Zheng, Yueqin Li

**Affiliations:** Key Lab of All Optical Network and Advanced Telecommunication Network of EMC, Beijing Jiaotong University, Beijing, 100044 China

## Abstract

We present a novel approach for triangular-shaped waveform generation by applying optical single sideband (OSSB) modulation and optical carrier suppression (OCS) modulation to an input signal. Firstly, an OSSB modulated signal consists of an optical carrier and +1st-order sideband, is initially generated with a dual-drive Mach-Zehnder modulator (DD-MZM1) driven by a quadruple frequency RF signal. By tuning the amplitude of the RF signal and the bias of the DD-MZM1, the power ratio between the carrier and the +1st-order sideband is controlled as 19 dB. These two components are then transmitted to a DD-MZM2 which is driven by a fundamental frequency. After OCS modulation, four sidebands are existed in optical spectra of the modulated signal. By utilizing a fiber Bragg grating (FBG) to remove the undesired sideband, an output signal that features a triangular-shaped waveform is finally achieved.

## Introduction

In recent years, photonic generation of arbitrary waveform signal is an attractive research area in microwave photonic. Among various signal profiles, signals with a triangular-shaped waveform, which is featured by linearly up-and-falling edges in time domain, has been widely used in all-optical data processing, and communication systems^1–3^. For example, optical triangular-shaped pump pulses can be used to covert time-division multiplexed (TDM) signals to wavelength-division multiplexed (WDM) ones^1^. Triangular-shaped pump pulses can also induce cross-phase modulation (XPM), which can be used in optical frequency conversion, pulse compression and signal copying^2,3^.

Traditionally, triangular-shaped waveforms are generated by electric methods but the generated signals have limitations like low carrier frequency and the narrow bandwidth^4^. With the development of photonic technology, these shortcomings in electronic technology could be effectively overcome. Therefore, photonic methods have emerged as a replacement because of the wide bandwidth, low loss and immunity to electromagnetic interference. One typical method is based on optical-spectrum-shaping combined with frequency-to-time mapping (FTTM)^5–7^. In this method, the spectral envelope of an optical frequency comb is modified by the spectral shaper to be a triangular spectral envelop, which can be mapped to the temporal waveform by using FTTM. The drawback of this kind of schemes is that the use of mode-locked laser (MLL) leads to a high cost and the generated triangular waveforms have small duty cycle (<1), which cannot satisfy the requirement in many applications where a full duty cycle (=1) is desired. To resolve this issue, generations based on external modulation of a continuous wave (CW) light using electro-optical modulators are proposed^8–16^. The basic principle of this kind of approach is to control the harmonics of optical intensity approximately equal to the first two-term Fourier components of a triangular waveform. In ref.^8^, we reported a triangular waveform generator based on spectrum manipulation. With the modulation of a single-drive Mach-Zehnder modulator (SD-MZM), five primary even-order sidebands are firstly generated. An optical interleaver followed by a chirped fiber grating (CFBG) is further employed to tune the power of modulation sidebands. Expression corresponding to the first two-term Fourier expansion of a triangular-shaped waveform is obtained in optical intensity. In ref.^9,10^. Liu *et al*. proposed a method to generate triangular waveform using a MZM biased at the null point and stimulated Brillouin scattering (SBS) in optical fiber. However, the SBS effect is sensitive to the environment, which consequently weakens the system stability. Triangular waveforms can also be obtained by using a MZM combined with an optical interleaver^11^ or a dispersive element^12,13^. The structure based on interleaver suffers from stability problem, and structure based on dispersive fiber suffers low tenability of repetition rate of the generated waveform. In addition to the approaches mentioned above, triangular waveform has also been obtained by using frequency comb generation and optical spectrum manipulation^14^, and time-domain synthesis^15,16^. Through these approaches, it can be seen that external modulation could effectively reduce the complexity of operation.

In this paper, we propose a novel photonic approach to generate triangular-shaped waveform signals based on two cascaded DD-MZMs. A signal consisting of a carrier and a sideband is firstly generated by the OSSB modulation in DD-MZM1. The frequency interval of the two components is 4 *f*_*m*_, where *f*_*m*_ is the frequency of RF signal from local oscillator (LO). Through adjusting the modulation index of DD-MZM1 properly, the power ratio of the two components is well controlled to 19 dB. With the optical carrier suppressed (OCS) modulation provided by the second modulator (DD-MZM2), each component is further split into two new ones as long as modulation index of DD-MZM2 is within a proper range. Thus four sidebands with a frequency interval of 2 *f*_*m*_ are existed in optical spectrum. After removing the undesired sideband by a fiber Bragg grating (FBG), optical intensity with an expression corresponding to the Fourier expression of an ideal triangular-shaped waveform is obtained. We have successfully generated a 10 GHz triangular-shaped waveform signal with a 5 GHz RF driving signal.

## Results

### Design of triangular-shaped waveform generator

**Figure 1:**
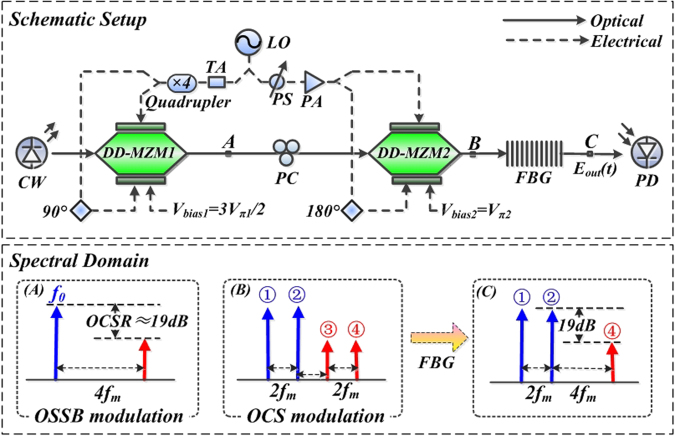
(**A**) Schematic diagram of the proposed triangular-shaped waveform generator; (**B**) The corresponding spectrum diagram. (CW, continuous-wave laser; LO, local oscillator; DD-MZM, dual-drive Mach–Zehnder modulator; TA, tunable attenuator; PS, phase shifter; PA, power amplifier; PC, polarization controller; FBG, fiber Bragg grating; PD, Photodetector).

The schematic setup of the proposed triangular-shaped waveform generation is shown in Fig. 1(A). A lightwave with an angular frequency of *ω*_0_ from the continuous wave (CW) laser is coupled into DD-MZM1 to perform the OSSB modulation. A radio frequency (RF) signal with a frequency of *ω*_*m*_ and amplitude of *A*_*m*_ is divided into two paths by a power divider. One path transmitted through a quadrupler is used to drive the DD-MZM1. The driving RF signal can be expressed as *A*_*rf1*_(*t*) = *A*_*m1*_exp(j4*ω*_*m*_*t*), which is then split into two parts by a 90° electric bridge to drive the two ports of DD-MZM1. The bias voltage of the DD-MZM1 is set to be *V*_*bias1*_. The optical field at the output of DD-MZM1 (point A in Fig. 1) can be generally expressed as:

1 $$\begin{array}{c}{E}_{A}(t)={E}_{in}(t)\{\begin{array}{c}\exp [j{\beta }_{1}\,\cos (4{\omega }_{m}t)]\\ +\,\exp [j{\beta }_{1}\,\cos (4{\omega }_{m}t+\frac{\pi }{2})+j\frac{{V}_{bias1}}{{V}_{\pi 1}}\pi ]\end{array}\}\\ \quad \quad \,\,=\,{E}_{in}(t)\sum _{n=-\infty }^{\infty }{k}_{n}\exp [jn(4{\omega }_{m})t]\end{array}$$

where *E*_*in*_(*t*) = *E*_0_exp(j*ω*_0_*t*) is the optical field at the input of the DD-MZM1 and ${\beta }_{1}=(\pi {A}_{m1})/(\sqrt{2}{V}_{\pi 1})$ is defined as the modulation index with *V*_*π*1_ referring to the half-wave switching voltage of DD-MZM1. In Eq. (1), *k*_*n*_ represents the weight value of the n_th_-order sideband,

2 $${k}_{n}={(\frac{1}{\sqrt{2}})}^{n}[{j}^{n}+{(-1)}^{n}\exp (j\frac{{V}_{bias1}}{{V}_{\pi 1}}\pi )]\,{J}_{n}({\beta }_{1})$$

*J*_*n*_(*·*) represents the Bessel function of the first kind of order n.

### Optical single sideband modulation

In our scheme, the OSSB modulation technique consists of a 90° electric bridge and a DD-MZM. By adjusting the bias voltage of the DD-MZM1 properly, optical lower (*V*_*bias1*_ = *V*_*π*_/2) or upper (*V*_*bias1*_ = 3*V*_*π*_/2) sideband modulation can be realized. With a small signal modulation, the DD-MZM1 is under a weakly modulated mode. In this case, the impact of the higher-order harmonic (nå 1) is negligible^17^. Here, when the bias point of the DD-MZM1 is biased at *V*_*bias1*_ = 3*V*_*π1*_/2, a signal with optical carrier and + 1st-order sideband in optical spectrum is achieved as shown in Fig. 1(A). Thus the output optical field after OSSB modulation can be simplified as:

3 $${E}_{A}(t)={E}_{0}\{{k}_{0}\exp (j{\omega }_{0}t)+{k}_{+1}\exp [j{\omega }_{0}t+j(4{\omega }_{m}t)]\}$$

*OCSR* is defined as the optical carrier to sideband ratio, and it can be calculated as

4 $$OCSR=10{\mathrm{log}}_{10}\frac{{|{k}_{0}|}^{2}}{{|{k}_{+1}|}^{2}}=10{\mathrm{log}}_{10}[{|\frac{{J}_{0}({\beta }_{1})}{{J}_{1}({\beta }_{1})}|}^{2}]$$

**Figure 2:**
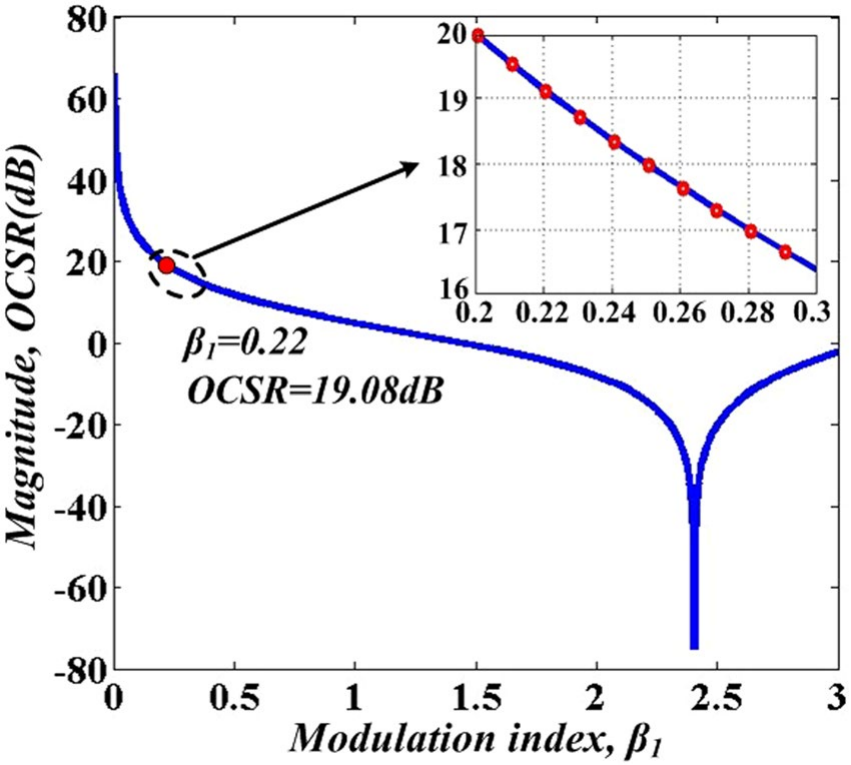
The OCSR (in decibels) versus modulation index, *β*_*1*_.

Figure 2 illustrates the relationship of *OCSR* versus modulation index, *β*_1_. By adjusting *β*_*1*_ within a range from 1 to 3, we can achieve an *OCSR* with continuous adjustment characteristics. As seen from the zoom-in picture in Fig. 2, when *β*_1_ is set to be 0.22, *OCSR* turns to be 19.08 dB, which means that optical carrier and +1st-order sideband with a power ratio of 19.08 dB is achieved. It can be seen that after OSSB modulation, two dominant optical components with an *OCSR* of 19.08 dB and a frequency interval of 4 *f*_*m*_ are existed in the spectrum, as shown in Fig. (A) in Fig. 1.

### Optical carrier suppression modulation

**Figure 3:**
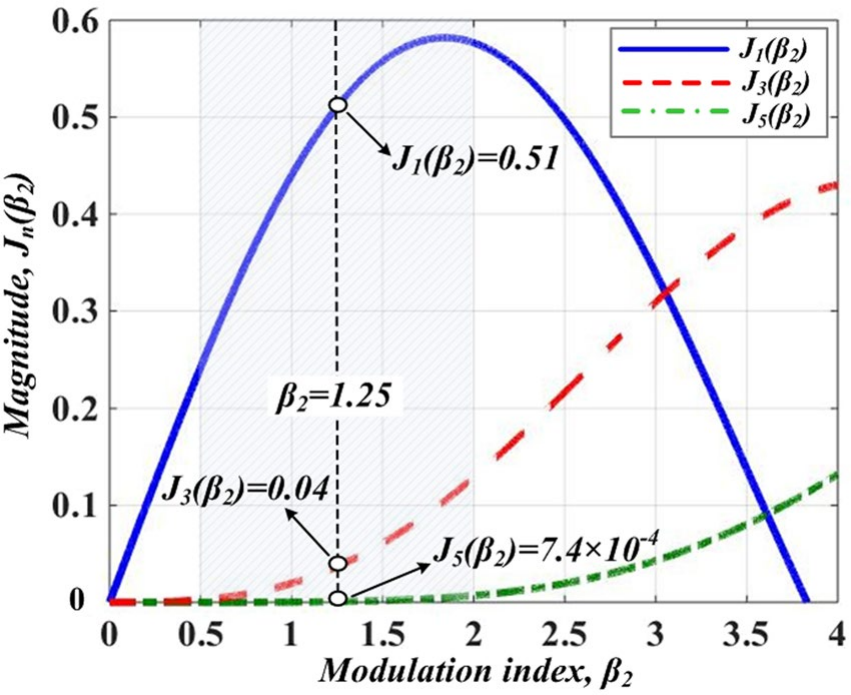
Bessel function *J*_*n*_ (·), versus modulation index, *β*_2_.

The OSSB modulated signal with two components is coupled into DD-MZM2 to perform the OCS modulation subsequently. The other path of RF signal from LO, expressed as *A*_*rf*2_*(t)* = *A*_*m2*_*exp(jω*_*m*_*t* + *φ)*, is divided into two parts with a phase difference of *π* to drive the two ports of DD-MZM2. As in Fig. 1, the amplitudes (*A*_*m2*_) can be adjusted by a tunable attenuator (TA) and a power amplifier (PA). A phase shifter (PS) is applied to ensure the coherence of the two paths of the RF signals from LO. A polarizer controller (PC) is used to minimize the polarization-dependent losses of the two DD-MZMs. The bias point of the DD-MZM2 is set to be *V*_*bias2*_, which is biased at the minimum point (*V*_*bias2*_ = *V*_*π2*_). After these adjustments, OCS modulation in realized, which means optical carrier and even-order sidebands are suppressed. The modulation index is calculated as *β*_*2*_ = *πA*_*m2*_/*V*_*π*_. Figure 3 gives the relationship between *β*_2_ and *J*_*n*_(·). Note that when *β*_2_ is adjusted within a range from 0.5 to 2, *J*_3_*(β*_2_) and *J*_5_*(β*_2_) are negligibly small. Thus, only ±1st-order sidebands are considered in the OCS modulation processing.

Since a modulator with a relatively small modulation index is more practical, we take *β*_*2*_ = 1.25 for example. In this case, *J*_1_*(β*_2_*)*, *J*_3_*(β*_2_) and *J*_5_*(β*_2_) turn out to be 0.51, 0.04 and 7.4 × 10^−4^ respectively, which shows that *J*_3_*(β*_2_) and *J*_5_*(β*_2_) are far smaller than *J*_1_*(β*_2_). This means that we only consider the ±1st-order optical sidebands in OCS modulation processing. Thus after OCS modulation, four optical sidebands with a frequency interval of 2 *f*_*m*_ exist in the optical spectrum. The spectrum diagram is as shown in Fig. 1(B), which consists four frequency components of *f*_0_ − *f*_*m*_, *f*_0_ + *f*_*m*_, *f*_0_ + 3 *f*_*m*_, and *f*_0_ + 5 *f*_*m*_ (corresponding to sidebands①, ②, ③ and ④, respectively). The optical field at the output of DD-MZM2 (point B) can be expressed as

5 $${E}_{B}(t)\propto \{\begin{array}{c}{k}_{0}[\exp (j{\omega }_{0}t-j{\omega }_{m}t)+\exp (j{\omega }_{0}t+j{\omega }_{m}t)]\\ +\,{k}_{+1}[\exp [j{\omega }_{0}t+j(3{\omega }_{m}t)]+\exp [j{\omega }_{0}t+j(5{\omega }_{m}t)]]\end{array}\}$$

Then a fiber Bragg grating (FBG) is employed to remove the undesired sideband ③, after which the output signal (point C in Fig. 1) only consists of sidebands ①, ② and ④. The optical field of the signal at the output of FBG can be expressed as

6 $${E}_{C}(t)\propto \{\begin{array}{c}{k}_{0}\exp (j{\omega }_{0}t-j{\omega }_{m}t)+{k}_{0}\exp (j{\omega }_{0}t+j{\omega }_{m}t)\\ +\,{k}_{+1}\exp [j{\omega }_{0}t+j(5{\omega }_{m}t)]\end{array}\}$$

The corresponding optical intensity can be calculated as

7 $${I}_{C}(t)\propto {I}_{dc}+{k}_{0}^{2}\,\cos (2{\omega }_{m}t)+{k}_{+1}^{2}\,\cos (6{\omega }_{m}t)$$

It is well known that a Fourier expansion of typical triangular-shaped waveform is given by

8 $${T}_{tr}(t)\propto \sum _{i=1}^{\infty }\frac{1}{{(2i-1)}^{2}}\cos [(2i-1){\rm{\Omega }}t]=\,\cos ({\rm{\Omega }}t)+\frac{1}{9}\,\cos (3{\rm{\Omega }}t)+\frac{1}{25}\,\cos (5{\rm{\Omega }}t)+\cdots $$

**Figure 4:**
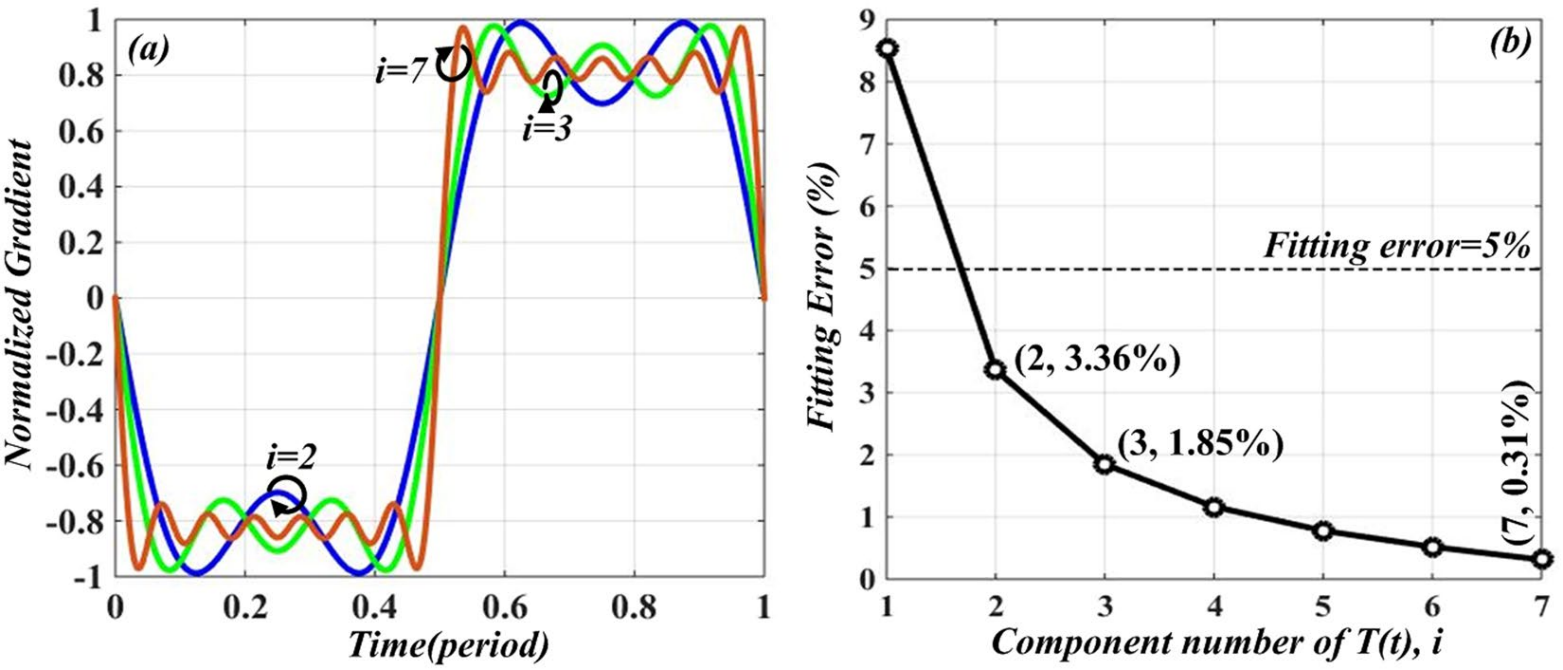
(**a**) Calculated normalized gradient of *T*_*tr*_*(t)* at *i* = 2, 3 and 7. (**b**) Relationship between fitting error *η* and component number of *T*_*tr*_*(t)*.

Obviously, Fourier expansion of an ideal triangular-shaped waveform signal contains infinite components. However, in the practical process of generating triangular-shaped waveform signal, we can only use finite components. Figure 4(a) gives the calculated gradients of *T*_*tr*_*(t)* at different *i* = 2, 3 and 7, where *i* denotes the component number of *T*_*tr*_*(t)*. Note that the gradients are approximately constant across the up-and-falling edges of the waveform when the coefficient *i* equals to 7. Since the energy of triangle-shaped waveform signal is concentrated on low frequency components and the attenuation of higher order components is fast, we can regard the influence of high order harmonics on the generated triangular waveform signal is negligibly small.

In order to explain this more intuitively, the parameter of fitting error *η* is defined as

9 $$\eta =\sqrt{\frac{{{\sum }_{i=1}^{i}({X}_{i}-{Y}_{i})}^{2}}{{\sum }_{i=1}^{i}({X}_{i}^{2}+{Y}_{i}^{2})}}\times 100 \% ,i=1,2,3,\ldots $$

where *X*_*i*_ is the amplitude of the *i*-th component of triangular-shaped waveform with finite components, and *Y*_*i*_ is the amplitude of the *i*-th component of the ideal triangular-shaped waveform signal [*T*_*tr*_*(t)* in Eq. (8)]. The fitting error *η* can be used to verify the similarity of the generated waveform. Figure 4(b) illustrate the relationship between *i* and fitting error *η*. Generally, when the fitting error is small enough (less than 5%), the triangular-shaped waveform signal can be considered desirable. We can see from Fig. 4(b) that when *i* = *2*, fitting error *η* is 3.36%. Thus we can use the first two items in Eq. (8) to construct a triangular-shaped waveform.

In addition, the power ratio between *Ω* and 3*Ω* terms should be 1/9 (namely, 19.08 dB). Comparing the expression of *I*_*c*_*(t)* in Eq. (7) with triangular-shaped waveform expansion *T*_*tr*_(*t*) in Eq. (8), by substituting *Ω* = 2*ω*_*m*_, we can conclude that the desired harmonics in *I*_*c*_(*t*) can make good approximation with the first two terms in Eq. (8), only if ${k}_{0}^{2}/{k}_{1}^{2}=9$ (namely, *OCSR* = 19.08 dB) is satisfied. As in the previous discussion in Eq. 4 and Fig. 2, by tuning *β*_1_ to 0.22, *OCSR* = 19.08 dB is obtained. Here, a triangular-shaped waveform signal is achieved. It can also be found from Eq. (7) that the repetition rate (RR) of the generated triangular-shaped waveform signal is 2 *f*_*m*_, which is twice of the driving frequency *f*_*m*_. Figure 1(A–C) illustrate the evolution of optical spectrum at points A, B and C.

## Methods

### Generation of Triangular-shaped waveform

OptiSystem 10.0 is used to perform the simulations and verify the proposed scheme. The schematic setup is shown in Fig. 1. A CW laser works at a central wavelength of 1550 nm, a line-width of 0.8 MHz and output power of 10 dBm. DD-MZM1 and DD-MZM2 possess an insertion loss of 5.5 dB, half-wave switching voltage of 2 V and extinction ratio of 30 dB. The DC bias of DD-MZM1 is set to quadrature point (*V*_*bias1*_ = 3*V*_*π*1_/2) and that of DD-MZM2 is set to minimum point (*V*_*bias2*_ = *V*_*π2*_). In our case, taking the driving frequency *f*_*m*_ = 5 GHz for example. The modulation index *β*_*1*_ is tuned to be 0.22 and *β*_2_ is set to be 1.25.

**Figure 5:**
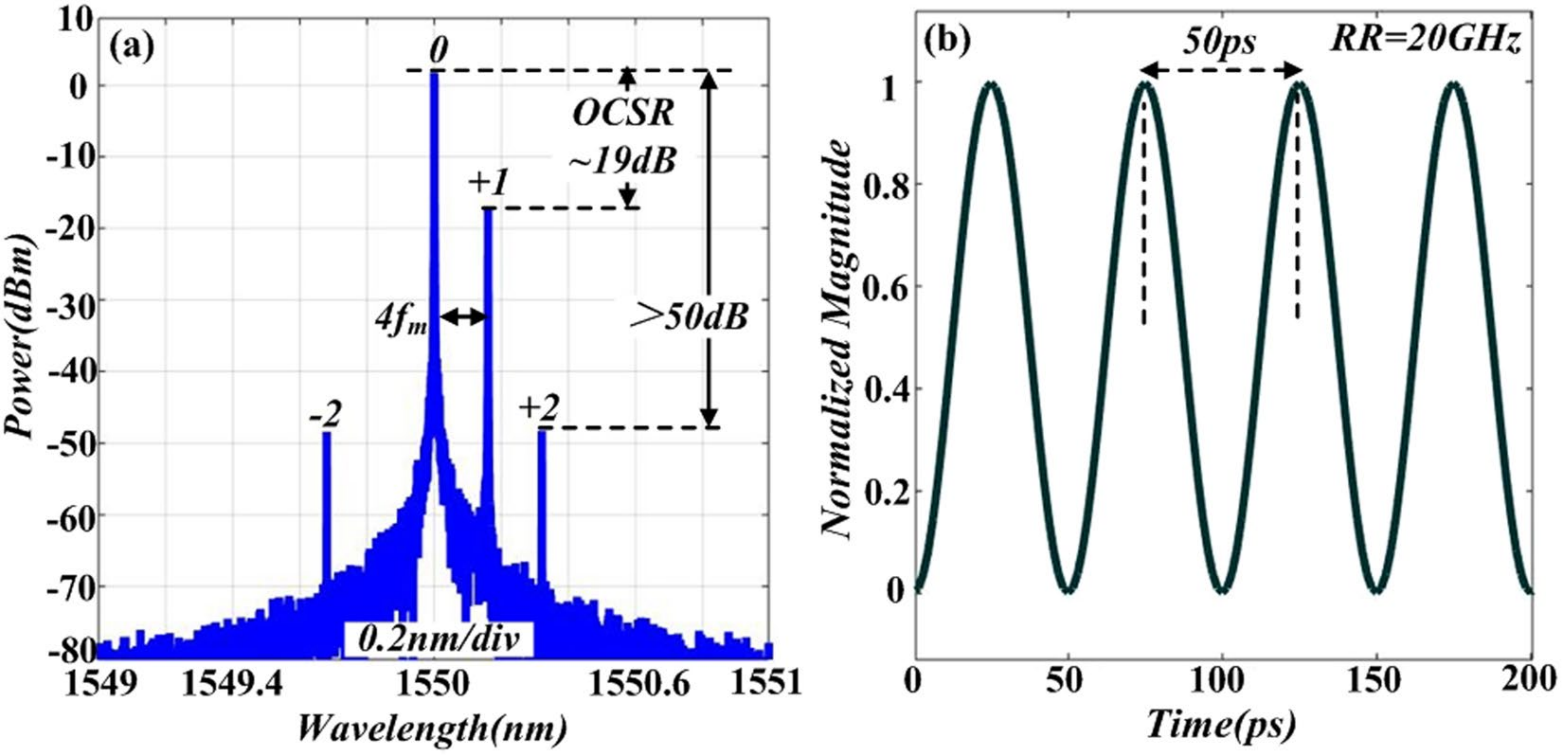
Simulated optical spectrum (**a**) and corresponding normalized intensity profile (**b**) of signal after DD-MZM1.

Pictures in Fig. 5 show the simulation results for optical spectrum and corresponding normalized intensity profile of OSSB modulation signal. It can be seen from Fig. 4(a) that after OSSB modulation, the power ratio between optical carrier and +1st-order sideband is 19 dB, while power of ±2nd-order sidebands is 50 dB lower than that of carrier. This means the power of sidebands higher than +1st-order is small enough to be neglected. Also note that the frequency interval between carrier and +1st-order sideband is 4 *f*_*m*_. Figure 5(b) shows the normalized intensity profile of the OSSB modulation signal. Here, the signal performs sinusoid-shaped intensity and the repetition rate (RR) is 20 GHz.

**Figure 6:**
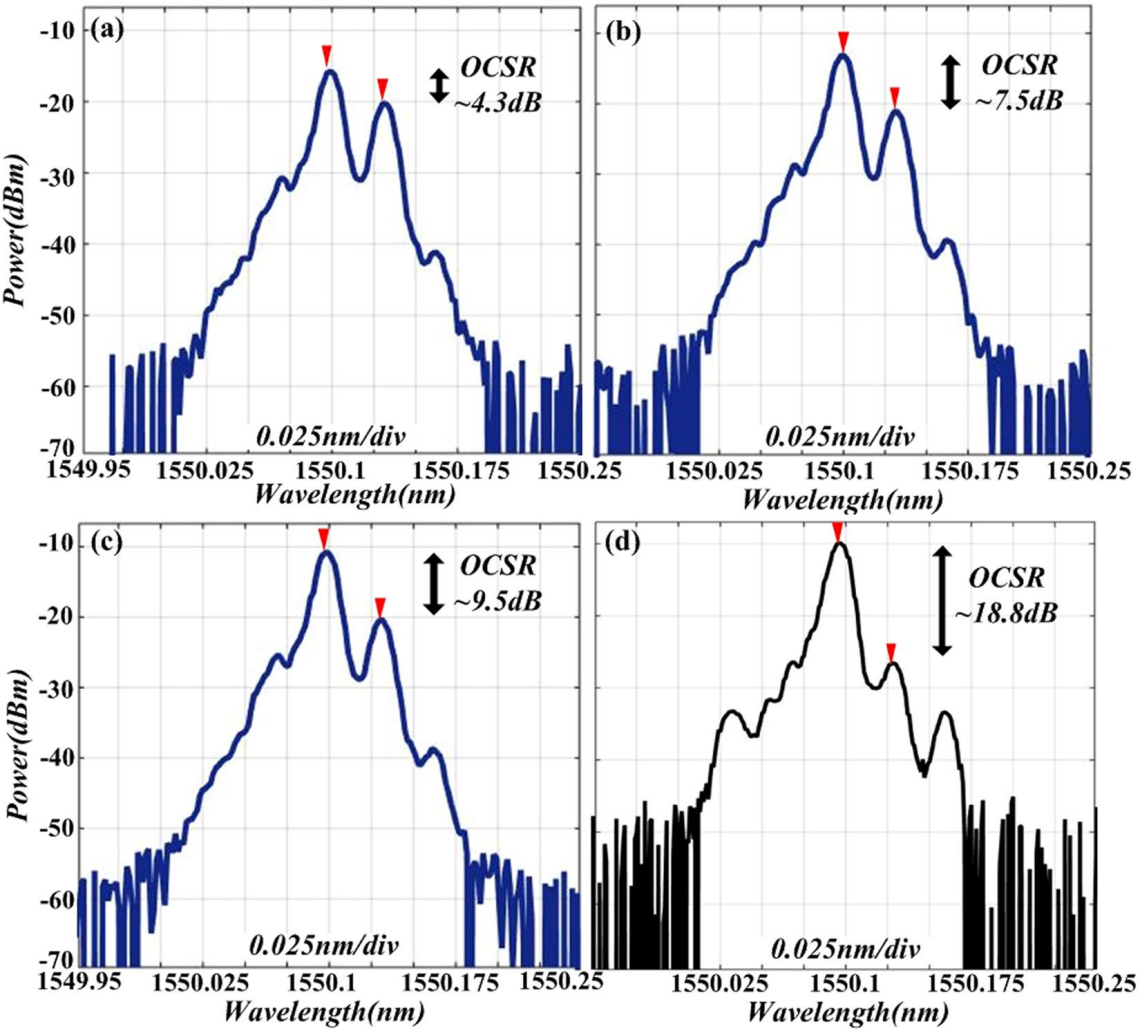
Experimental optical spectrum of OSSB modulation signal with different modulation index: (**a**) 1.05; (**b**) 0.78; (**c**) 0.64; (**d**) 0.22.

To further investigate the mechanism of the OSSB modulation, a proof-of-concept experiment is carried out. A tunable CW laser (Agilent 8164 A) works at a central wavelength of 1550.10 nm and power of 10 dBm. The RF signal generator (YIAI AV1431A) generates a 4 GHz sinusoid signal to act as the driving signal of DD-MZM1 (Fujitsu FTM7962EP). The optical spectrum with different modulation index is shown in Fig. 6, which is measured by an optical spectrum analyzer (Ando AQ6317C). As shown in Fig. 6(a–d), we experimentally performed optical spectrum with four different modulation index cases to verify the feasibility of the OSSB modulation in our scheme. When the applied modulation index *β*_1_ is around 1.05, 0.78, 0.64, and 0.22, the OCSR prove to be 4.3 dB, 7.5 dB, 9.5 dB and 18.8 dB, respectively.

Based on the experimental results, a conclusion can be drawn that the *OCSR* can be continuously tuned by applying different modulation index, which satisfies with the theoretical result of relationship between *OCSR* versus modulation index *β*_1_ in Fig. 2. Specially, when modulation index *β*_1_ is tuned to 0.22, the *OCSR* is 18.8 dB (close to 19 dB) and the frequency interval between carrier and +1st-order sideband is 4 GH, which accords with the previous discussion in Fig. 5. This satisfies the basic requirement of the generation of a triangular-shaped waveform signal.

**Figure 7:**
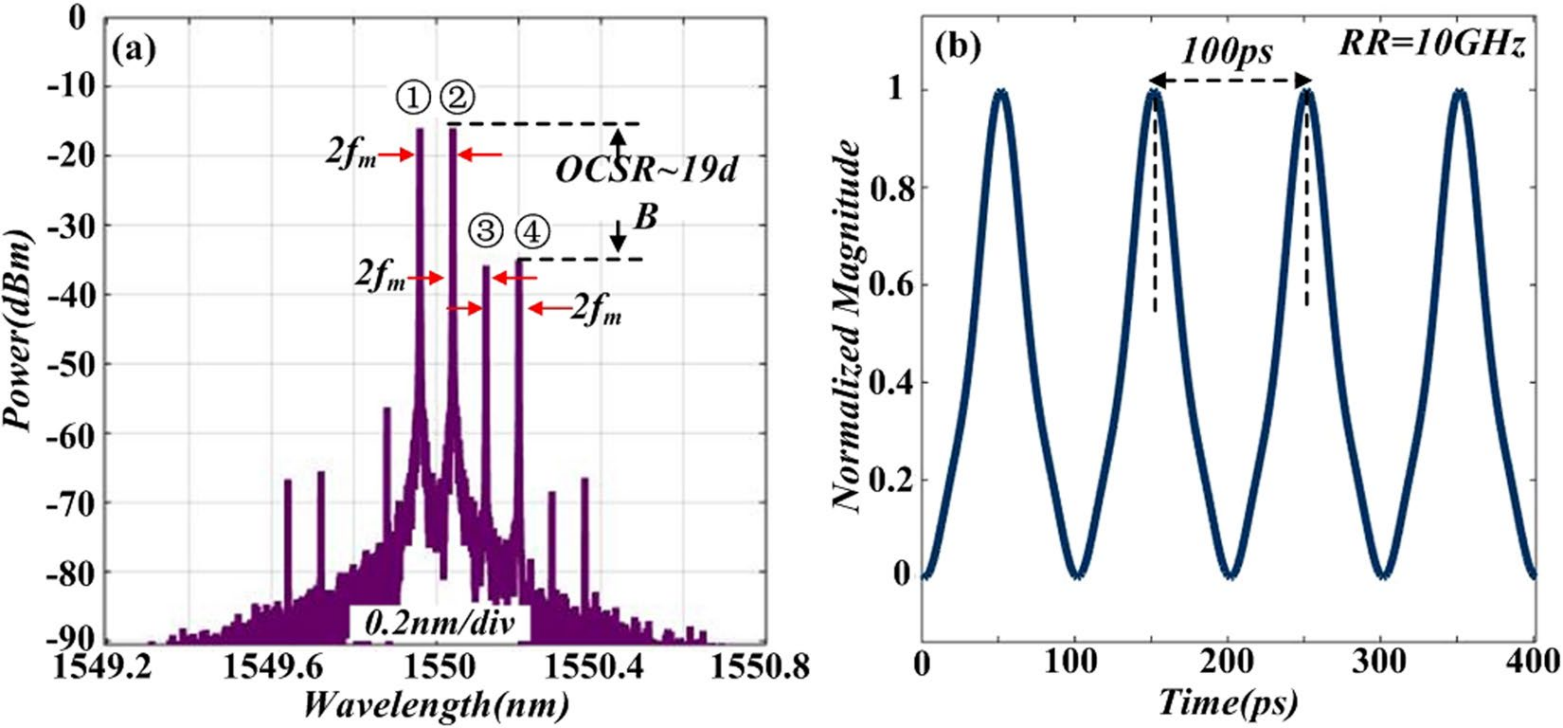
Simulated optical spectrum (**a**) and corresponding normalized intensity profile (**b**) of signal after DD-MZM2.

After OSSB modulation, DD-MZM2 is followed to implement the OCS modulation. Pictures in Fig. 7 shows the optical spectrum and normalized intensity profile of the signal after OCS modulation. Note that by tuning the *β*_2_ properly (*β*_2_ = 1.25 for example), undesired even-order sidebands are well suppressed. As a small signal modulation is implement, only ±1st-order sideband of OCS modulation is considered. Thus there exist four sidebands (①, ②, ③ and ④) in the optical spectrum at output of DD-MZM2, as shown in Fig. 7(a). The power ratio between sideband ② and sideband ④ is roughly19 dB and the frequency interval is 2 *f*_*m*._ Figure 7(b) shows the temporal waveform of the OCS modulation signal. The RR of the signal is 10 GHz, which is twice of the driving frequency of DD-MZM2. Obviously, the waveform in optical intensity is not triangular-shaped. This is because of the distortion induced by sideband ③. In the following step, we focus on removing the undesired sideband.

**Figure 8:**
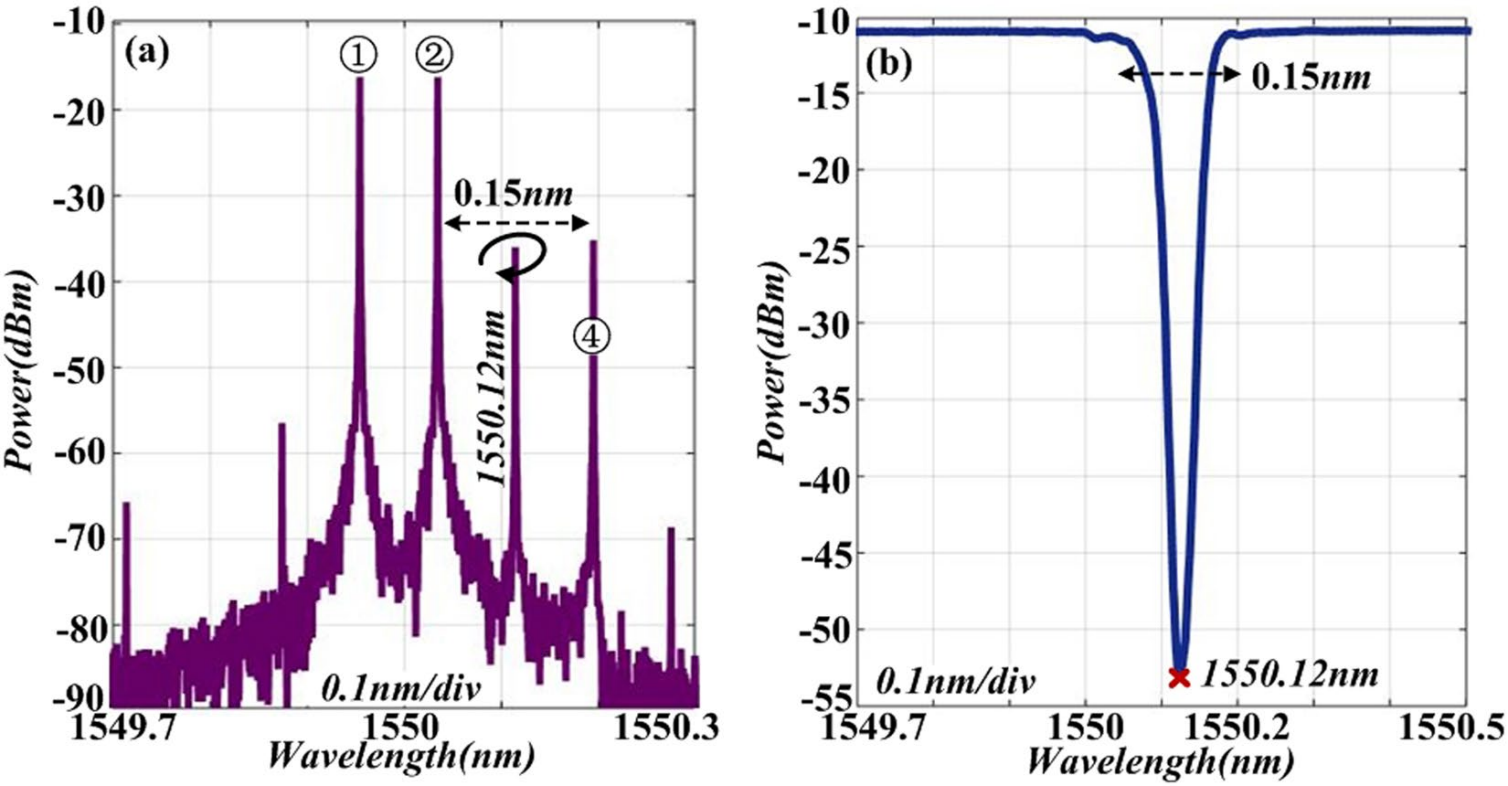
(**a**) The zoom-in optical spectrum of signal after DD-MZM2; (**b**) Experimental transmission spectrum of the FBG.

A piece of FBG is employed to remove the interfering sideband ③. Figure 8(a) gives the zoom-in optical spectrum of signal after DD-MZM2. As can be seen that the wavelength of sideband ③ is 1550.12 nm and spacing between sideband ② and ④ is 0.15 nm. We fabricate a piece of FBG and obtain its transmission spectrum, as shown in Fig. 8(b). The transmission depth of around 40 dB and block width is 0.15 nm. After FBG, the power of undesired sideband ③ can be suppressed under −55 dBm. Obviously, the FBG can be used to obtain the desired spectrum.

**Figure 9:**
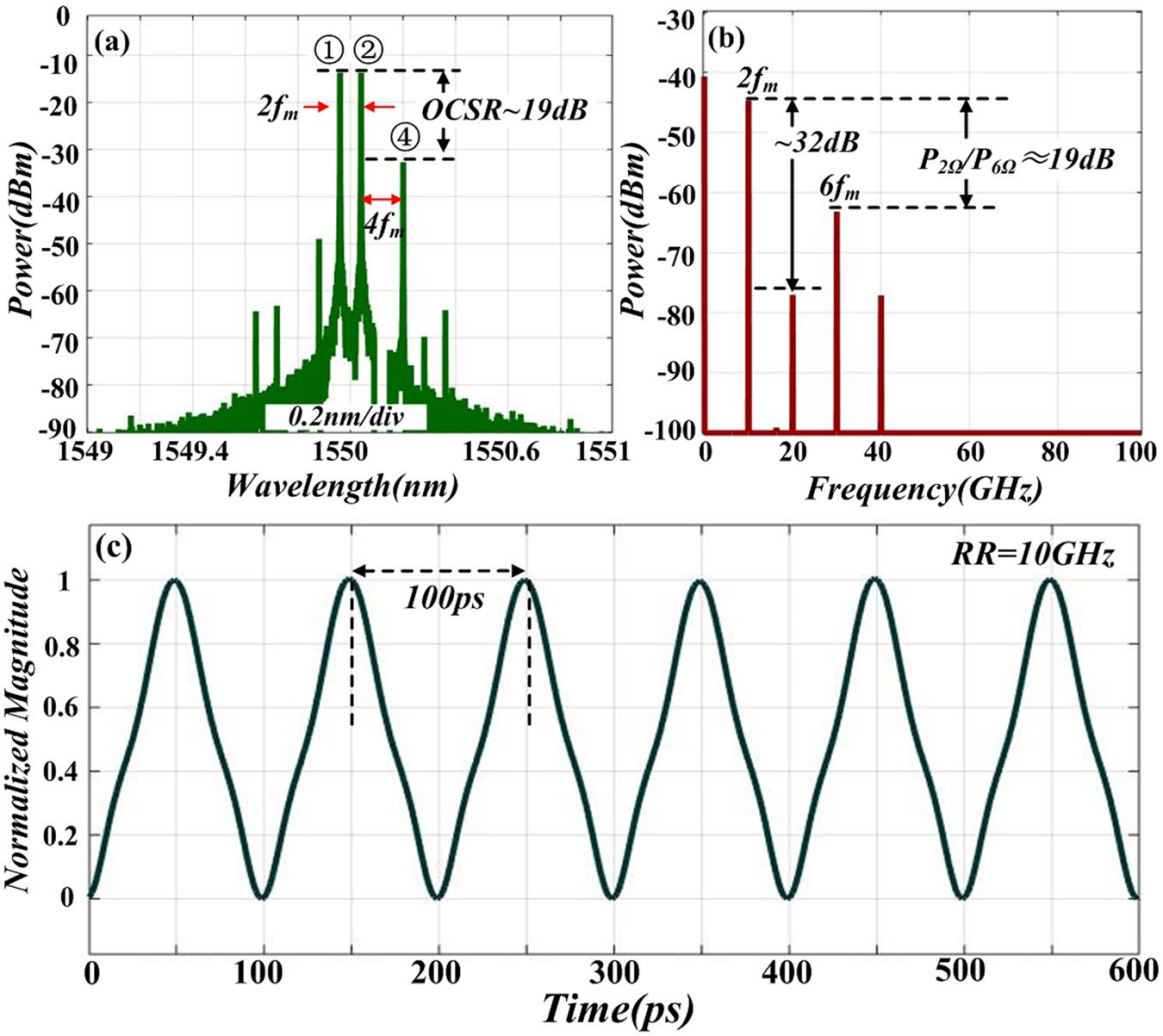
(**a**) Optical spectrum of the generated signal; (**b**) The corresponding electrical spectrum; (**c**) Temporal waveform of triangular-shaped waveform.

Figure 9(a) plots the output optical spectrum after FBG. Note that after filtering, only three major sidebands exist in the spectrum (①, ②, and ④). The power ratio between sidebands ② and ④ is 19 dB, which is in accordance with the character of a triangular-shaped waveform signal. To further illustrates the characteristic of the generated signal, electrical spectrum is also carried out. Figure 9(b) gives the simulated electrical power spectrum of the generated signal, in which the power ratio between the 10 and 30 GHz components (P_2Ω_/P_6Ω_) is 19 dB, indicating that the amplitude of the first-order harmonic is nine times of that of the third-order harmonic. This also agrees well with the property of a triangular-shaped waveform signal. In addition, note that the second-order harmonic (20 GHz component) is 32 dB lower than the first-order harmonic. This means the impact of the undesired harmonic has been well suppressed. The normalized intensity profile of the generated signal is given in Fig. 9(c). It is obvious that a triangular-shaped waveform is obtained in the optical intensity. The pulse repetition is double of the driving frequency and the pulse duration is 500 ps.

**Figure 10:**
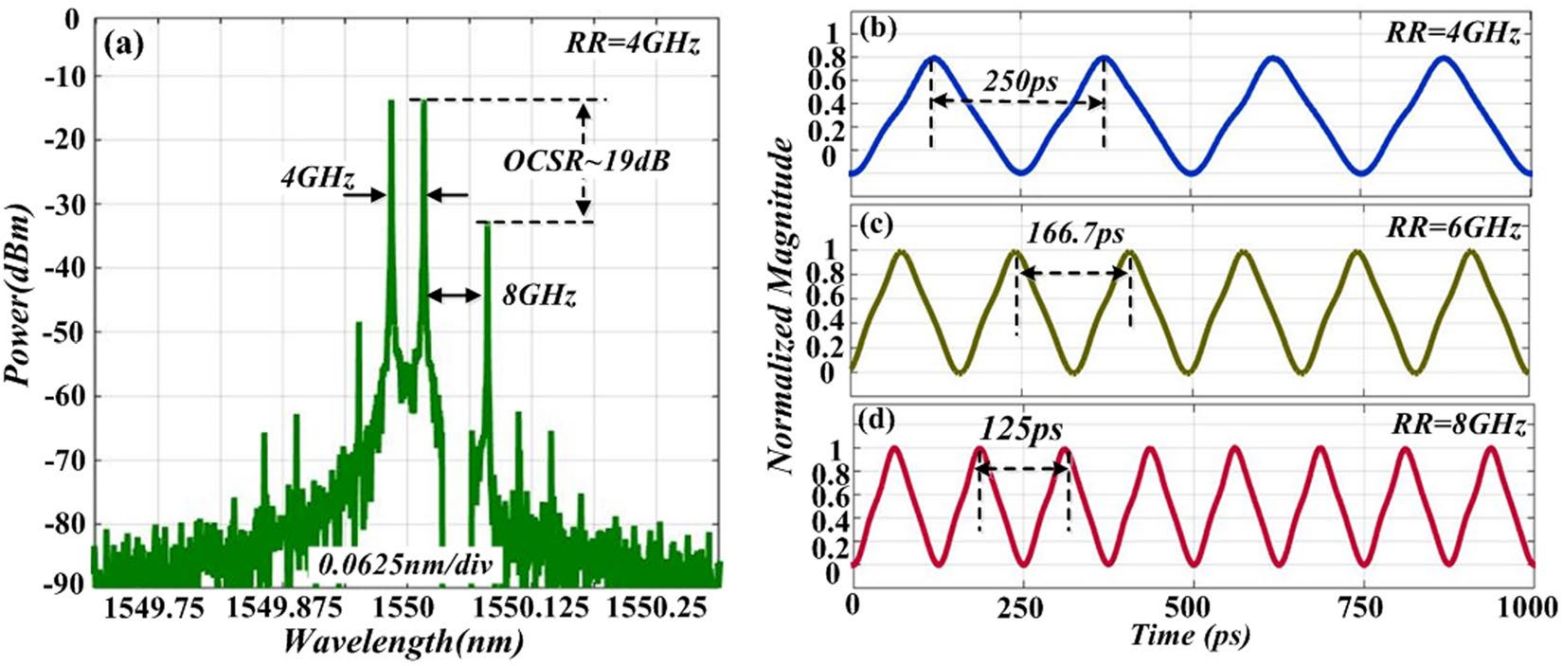
(**a**) Optical spectrum of the 4 GHz triangular-shaped waveform signal; Simulated temporal waveform of optical intensity with repetition of: (**b**) 4, (**c**) 6, (**d**) 8 GHz.

Results in Fig. 9 prove that after OSSB modulation, OCS modulation and filtering out interfering sideband, a 10 GHz triangular-shaped waveform signal is acquired by using a 5 GHz driving RF signal. In order to investigate the feasibility and tunability of the proposed scheme, we simulated the generation of triangular-shaped waveform signals with different repetition rate (RR). Figure 10(a) and (b) plot the optical spectrum and corresponding temporal waveform of the generate triangular-shaped waveform signal with RR of 4 GHz. In this case, the power ratio between sidebands ② and ④ keeps 19 dB, which satisfies the characteristic of a triangular-shaped waveform signal. Figure 10(c,d) show the temporal waveform of *I*_*out*_*(t)* at two different repetition rate (6 and 8 GHz) by tuning driving frequency to 3 GHz and 4 GHz respectively. This indicates that the generated triangular-shaped waveform signal has tunable repetition rate which is double of the driving frequency. Ultimately, even if low driving frequency is applied, waveform signal with high repetition rate could still be generated, which expands the tuning range of the optical triangular-shaped waveform signal.

### Analysis of bias point drifts of DD-MZMs

**Figure 11:**
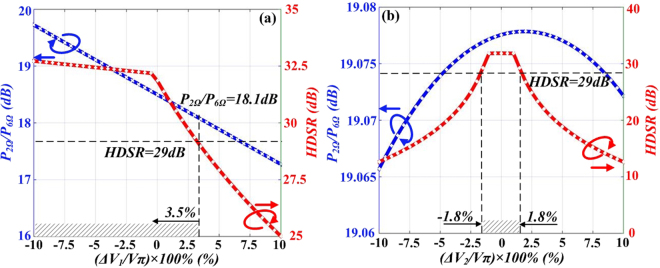
Simulated *P*_*2Ω*_*/P*_*6Ω*_ and *HDSR* curves versus MZM bias voltage: (**a**) Bias drift of DD-MZM1, (**b**) Bias drift of DDMZM2.

In the previous discussion, two DD-MZMs are biased at quadrature point and MITP with no bias drifts. Practically, the influence of the bias drifts must be considered because they may have impact on the stability and quality of the generated triangular-shaped waveform signal. Harmonic distortion suppression ratio (*HDSR*) is defined as the power ratio of 1st-order harmonic and the primary interfering harmonic, and *P*_*2Ω*_*/P*_*6Ω*_ is defined as the power ratio of 2nd- and 6th-order harmonic. We focus on these two parameters to evaluate the influence of the bias drifts on generated triangular-shaped waveform signal. The bias drift can be expressed as *ΔV*_*bias*_ = (*ΔV/V*_*π*_) × 100%, *ΔV*_*bias1*_ and *ΔV*_*bias2*_ denote the bias of DD-MZM1 and DD-MZM2 respectively. Pictures in Fig. 11 illustrate the numerical simulations about *P*_*2Ω*_*/P*_*6Ω*_ and *HDSR* versus *ΔV*_*bias1*_ and *ΔV*_*bias2*_ changing within a certain range. Assuming that when HDSR is higher than 29 dB and P_2Ω_/P_6Ω_ is within a range of 18 dB to 20 dB, the undesired distortion is acceptable. In Fig. 11(a), when *ΔV*_*bias1*_ varies from −10% to +10%, *P*_*2Ω*_*/P*_*6Ω*_ decreases from 19.8 dB to 17.4 dB and HDSR decreases from 32.5 dB to 25 dB. When HDSRå 29 dB and 19.8 dB < *P*_*2Ω*_*/P*_*6Ω*_ < 17.4 dB is required, the tolerable range of the bias drift of DD-MZM1 proves to be −10% ≤ *ΔV*_*bias*_ ≤ 3.5%. Figure 11(b) shows *P*_*2Ω*_*/P*_*6Ω*_ and *HDSR* curves versus *ΔV*_*bias2*_. As shown in the figure, *P*_*2Ω*_*/P*_*6Ω*_ almost remains constant to 19.08 dB while *ΔV*_*bias2*_ varies from −10% to +10%. This indicates that the bias drift of DD-MZM2 has limited influence on *P*_*2Ω*_*/P*_*6Ω*._ Within this range, HDSR decreases from 32 dB to 13 dB, which means that *HDSR* is sensitive to *ΔV*_*bias2*_. When HDSR is higher than 29 dB, the tolerable range of *ΔV*_*bias2*_ is −1.8% ≤ *ΔV*_*bias3*_  ≤ 1.8%.

**Figure 12:**
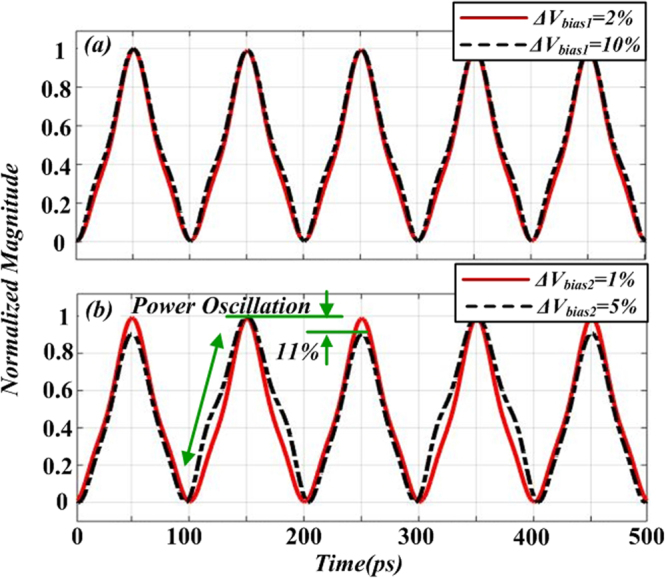
Temporal waveform of *I*_*out*_(t) with different bias drift cases: (**a**) bias drift of DD-MZM1, (**b**) bias drift of DDMZM2.

Figure 12 gives the temporal waveforms of *I*_*out*_*(t*) in different bias drift cases. The solid line in Fig. 12(a) shows the temporal waveform with bias drift of *ΔV*_*bias1*_ = 2% and the dotted line shows the temporal waveform with bias drift of *ΔV*_*bias1*_ = 10%. It can be seen that the intensity profile of the generated waveforms is very close, which means the stability and quality of the generated signal is not so sensitive to the bias drift of the DD-MZM1. In Fig. 12(b), the solid line shows that when the bias drift of DD-MZM2 is within an acceptable range (*ΔV*_*bias2*_ = 1%, for example), the intensity profile of signal keeps triangular-shape and the power level of the waveform is roughly stable. When the bias drift turns to *ΔV*_*bias2*_ = 5%, the Power Oscillation of intensity profile is relatively high (11%), and up-and-falling edge of the triangular-shape waveform is not linearly any more. Accordingly, the bias drift may affect the performance of the generated triangular-shaped waveform signal, such as the not exactly identical peak power and the linearity of the up-and-falling edge of triangular-shape waveform. In practical, by using a modulator with a large half-wave voltage or bias-controlled circuit to stabilize the bias voltage within an acceptable range.

### Data availability

Data from OptiSystem 10.0 simulations are available upon request from the corresponding author. External modulation experiment conducted at Beijing Jiaotong University can be made available from the corresponding author upon reasonable request.

## Discussion

In conclusion, we proposed a photonic method for the generation of a full-duty cycle triangular-shaped waveform signal based on external modulation in two cascaded DD-MZMs. Firstly, an OSSB modulated signal with a carrier and a sideband featuring an OCSR of 19 dB is generated by tuning the modulation index of a modulator (DD-MZM1). With the OCS modulation in the second DD-MZM, the two dominant optical components are subsequently split into four sidebands. A FBG is further employed to remove one undesired sideband and three sidebands with properly controlled intensity distribution is kept to form the desired triangular-shaped waveform signal. We have successfully demonstrated the generation process of triangular-shaped waveforms with repetition of 4, 6, 8 and 10 GHz which verified the feasibility and tunability of the proposed scheme. In addition, the impact of the DD-MZM bias drift *V*_*bias*_ variation on the temporal intensity of the generated waveform, which is a critical factor that might affect the system performance in practical applications, has also been evaluated, showing great stability within a certain range. This present method may provide the potential to generate waveform with triangular-shaped intensity considering its simplicity and feasibility and pave the way for various applications.
